# Health-E-Call, a Smartphone-Assisted Behavioral Obesity Treatment: Pilot Study

**DOI:** 10.2196/mhealth.2164

**Published:** 2013-04-17

**Authors:** J Graham Thomas, Rena R Wing

**Affiliations:** ^1^Weight Control and Diabetes Research CenterDepartment of Psychiatry and Human BehaviorWarren Alpert Medical School of Brown University & The Miriam HospitalProvidence, RIUnited States

**Keywords:** obesity, behavior, weight loss, mobile phone, technology

## Abstract

**Background:**

Individual and group-based behavioral weight loss treatment (BWL) produces average weight loss of 5-10% of initial body weight, which improves health and wellbeing. However, BWL is an intensive treatment that is costly and not widely available. Smartphones may be a useful tool for promoting adherence to key aspects of BWL, such as self-monitoring, thereby facilitating weight loss while reducing the need for intensive in-person contact.

**Objective:**

The objective of this study was to evaluate smartphones as a method of delivering key components of established and empirically validated behavioral weight loss treatment, with an emphasis on adherence to self-monitoring.

**Methods:**

Twenty overweight/obese participants (95% women; 85% non-Hispanic White; mean age 53.0, SE 1.9) received 12-24 weeks of behavioral weight loss treatment consisting of smartphone-based self-monitoring, feedback, and behavioral skills training. Participants also received brief weekly weigh-ins and paper weight loss lessons.

**Results:**

Average weight loss was 8.4kg (SE 0.8kg; 9%, SE 1% of initial body weight) at 12 weeks and 10.9kg (SE 1.1kg; 11%, SE 1% of initial body weight) at 24 weeks. Adherence to the self-monitoring protocol was 91% (SE 3%) during the first 12 weeks and 85% (SE 4%) during the second 12 weeks.

**Conclusions:**

Smartphones show promise as a tool for delivering key components of BWL and may be particularly advantageous for optimizing adherence to self-monitoring, a cornerstone of BWL.

## Introduction

Overweight and obesity are highly prevalent conditions among the populations of developed countries [1], contributing to increased risk of disease [2] and behavioral disorders [3], and place a substantial burden on financial and health care systems [2,4]. Behavioral weight loss treatments (BWLs) [5], such as those developed for the Diabetes Prevention Program (DPP) [6] and LookAHEAD (Action for Health in Diabetes) trials [7], produce weight loss by teaching skills to build healthy eating and physical activity habits. These programs produce average weight loss of 7-10% of initial body weight, which are associated with clinically significant improvements in physical health, disease risk factors, and indicators of psychological well-being [8-12]. Despite the challenges of weight loss maintenance, BWLs have been shown to produce lasting improvements in health [13].

BWL is a highly intensive treatment typically delivered in 30-60 minute individual or group treatment sessions, conducted weekly over the course of several months [5]. These sessions are costly to provide and require a substantial investment of time and resources by the recipients. Thus, BWL is not widely available outside of research settings, leading to efforts to identify alternative modalities for BWL delivery that reduce costs and barriers to treatment. For example, BWL has been delivered via the Internet [14-17] and in community settings such as the YMCA [18]. The average weight loss in these trials was typically much lower than the 7-10% of initial body weight obtained via intensive in-person treatment conducted in research settings, often because of insufficient exposure to the intervention and/or poor adherence to core behavioral weight loss strategies such as self-monitoring [19,20].

Mobile phones are also beginning to be considered as a modality for BWL delivery [21]. Previous research using mobile phones relied primarily on text messaging to provide brief suggestions and reminders for healthy behavior change. Patrick et al obtained an average weight loss of 3.16% of initial body weight via an automated, interactive, 4-month text message-based weight loss intervention (compared to 1.01% in a control condition) in a study with 78 participants [22]. Using a similar text message-based approach, Happala et al obtained an average weight loss of 5.3% (SD 3.5, N=42) of initial body weight at 3 months and 6.1% (SD 5.1) at 6 months [23]. Again, the average weight loss achieved in these studies were less than the 7-10% loss obtained via intensive in-person treatment conducted in research settings.

Mobile phone technology continues to progress at a rapid pace and the advent of smartphones makes it possible to deliver BWLs in new and more sophisticated ways. Mobile smartphones have many of the same capabilities as traditional personal computers, such as a persistent Internet connection, the ability to run sophisticated software applications (ie, “apps”), and the ability to play audio and video files nearly instantly from the Internet. Smartphones are prevalent, especially among ethnic minorities. Current estimates indicated that 30% of Whites, 44% of African Americans, and 44% of Hispanics own a smartphone in the United States [24,25]. Commercial apps for weight loss are very popular. Producers of one weight loss application, LoseIt!, reported that their app has been downloaded over 12 million times from the time it was first offered in 2008 to October 2012 [26].

Smartphone applications are now being developed to target changes in weight-related health behaviors and conditions related to obesity such as diabetes, but many do not adhere to evidence-based practice and few have been tested [27]. A recent review of mobile phone interventions to increase physical activity and reduce weight found two studies in which smartphone-based interventions were tested [28]. The first study by Gasser et al studied aspects of interface design and usage patterns of a smartphone application aimed at promoting increased physical activity and consumption of fruits and vegetables using a simplified points system for self-monitoring and team-based social interaction [29]. The second study by Lee et al developed and pilot-tested a smartphone-based game that provided a personalized diet profile and promoted knowledge about nutrition [30]. Another review by Hebden et al described the development of four smartphone applications aimed at preventing weight gain in young adults by increasing physical activity and consumption of fruits and vegetables, and reducing consumption of fast food and sugar-sweetened beverages [31]. Chomutare et al reviewed commercially available smartphone applications for diabetes management, and found that many were not consistent with evidence-based recommendations for diabetes self-care [32].

Despite the popularity of smartphone technology, it has never been tested as a means of enhancing self-monitoring and delivering empirically validated BWL content and interventionist feedback in a formal weight loss program. The purpose of the Health-E-Call study was to determine whether key components of BWL such as self-monitoring, feedback, and skills training could be accomplished and potentially enhanced via smartphones, thereby reducing the need for intensive in-person treatment. Particular emphasis was placed on using the smartphone to enhance self-monitoring, given the importance of this skill for successful weight loss. Previous research has shown that use of an electronic handheld device for self-monitoring improved adherence to the self-monitoring protocol [33], the accuracy of self-monitoring [34], and improved weight loss in traditional BWLs [33]. The primary outcome measures of this study were weight loss and adherence to the self-monitoring protocol. We also measured self-reported satisfaction with the program.

Because this was one of the first studies in which smartphones were used for BWL delivery, brief weekly visits with a study interventionist were included in the protocol to obtain objective weights and to provide an opportunity to address any challenges with the smartphone technology. Brief paper weight loss lessons were provided to participants to ensure sufficient exposure to behavioral weight loss strategies.

## Methods

### Participants and Recruitment

Overweight and obese men and women with a body mass index (BMI) of 25-50 kg/m^2^ between the ages of 18 and 70 were recruited by an advertisement posted on the website of the Brown University and Miriam Hospital Weight Control and Diabetes Research Center (WCDRC). The advertisement mentioned of the use of a smartphone for weight loss. Other inclusion criteria included English language fluency and literacy, and an ability to attend weekly treatment visits at the WCDRC in Providence, Rhode Island. Exclusion criteria included any heart conditions that limited ability to participate in physical activity, chest pain, any cognitive or physical limitation that prevented the use of a smartphone, recent serious mental illness, a history of or current eating disorder, previous or planned bariatric surgery, use of weight loss medication, pregnancy or expected pregnancy within 6 months of participation, a plan to leave the geographical region during the study period, participation in a study at the WCDRC within the previous two years, or a weight loss of greater than 5% body weight in the 6 months prior to study enrollment.

Interested individuals responded to the online advertisement by calling a phone number to be screened for eligibility and schedule an in-person individual orientation session at the WCDRC where they were given more information about the study and enrolled. Upon completing informed consent procedures, participants’ height and weight were measured and baseline questionnaires were administered. Participants then used the smartphone-based intervention system for 12 weeks and attended weekly weigh-ins with a study interventionist where they were given supplementary paper weight loss lessons. Upon completing 12 weeks of treatment, participants were given the opportunity to enroll in an extended treatment program consisting of an additional 12 weeks of access to the smartphone-based intervention system and weekly weigh-ins, but no additional weight loss lessons. Participants were not told of the opportunity to participate in the second 12-week treatment until the end of the first 12 weeks. The program was divided into 2 contiguous 12-week periods because it was unknown if participants would be able to maintain engagement with the novel smartphone-based intervention system for a full 24 weeks. Objective weights were obtained weekly during clinic visits. Questionnaire measures were administered at baseline and at the end of each 12-week treatment period.

### Intervention

The Health-E-Call treatment protocol was designed to deliver key components of established and empirically validated behavioral weight loss treatment such as the DPP [6] and LookAHEAD [7]. These multidimensional programs achieved weight loss through a combination of diet and physical activity education and training in behavioral strategies (eg, stimulus control) delivered in group and individual sessions and paper lessons. The program also included self-monitoring in paper diaries with written interventionist feedback returned at the next treatment session, and in-person support and accountability from treatment staff. Whenever possible, smartphones were used in Health-E-Call to implement and enhance each of these treatment components.

The Health-E-Call treatment included a smartphone-based component, a minimal in-person component consisting primarily of brief weekly weigh-ins, and supplementary paper weight loss lessons. The smartphone-based component was the focus of the intervention, and was the primary means of intervention delivery. An Apple iPhone was required for participation. Participants who did not own an iPhone were given an iPhone 3GS for the duration of the study.

The smartphone-based treatment component was divided into 3 parts including self-monitoring, feedback (automated and human), and brief videos for education and skills training. Given that self-monitoring is the cornerstone of BWL, self-monitoring with feedback was the primary focus on the smartphone-based treatment component. In this study, two separate smartphone applications, one developed by the research team (the Health-E-Call application), and one commercially available self-monitoring tool (DailyBurn) were used for self-monitoring, feedback, and delivery of video weight loss lessons.

**Figure 1:**
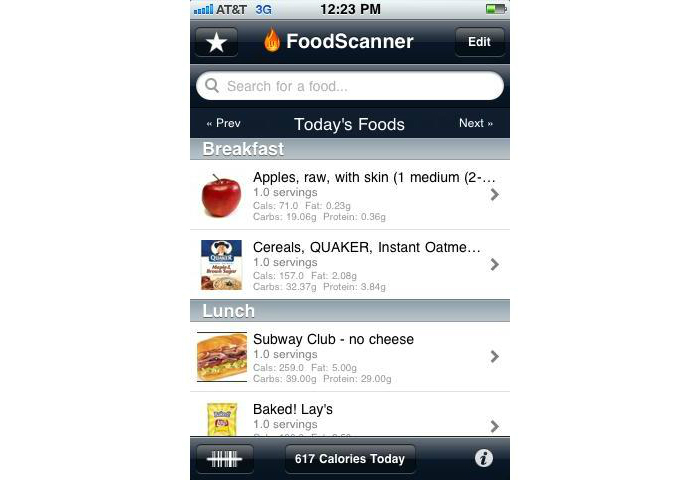
Self-monitoring of food intake via the DailyBurn application.

The commercially available DailyBurn smartphone application was used for self-monitoring of daily food intake, physical activity, and body weight (Figure 1). Compared to traditional paper diaries, this program simplified self-monitoring by allowing participants to record their intake by searching for foods by name/description or by scanning barcodes on food packages. Participants were also able keep a list of favorite foods for faster entry. A simple touch interface allowed the participant to indicate the quantity of the foods consumed and the application maintained a real-time total of calories and fat grams consumed, as well as other characteristics of the diet. Similar procedures were used to record bouts of physical activity and daily body weight. The Health-E-Call team had no contact with DailyBurn prior to, or during the study. However, the intervention team was able access participants’ responses in real-time via a system developed by the first author to automate retrieval of data from DailyBurn using participants’ login credentials.

**Figure 2:**
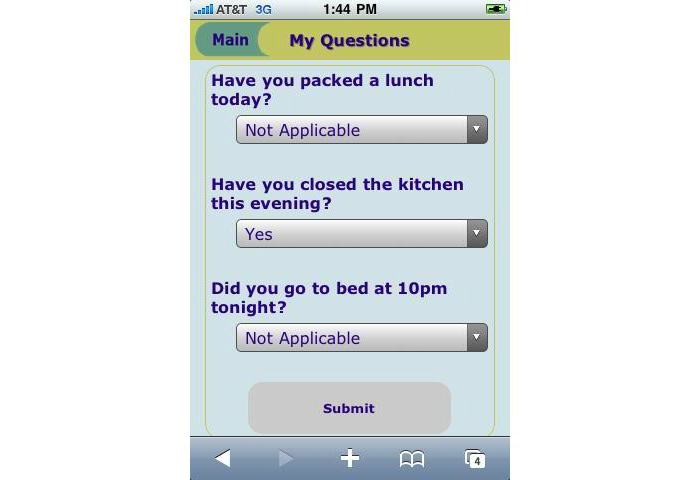
Self-monitoring of personalized behaviors via the Health-E-Call application.

**Figure 3:**
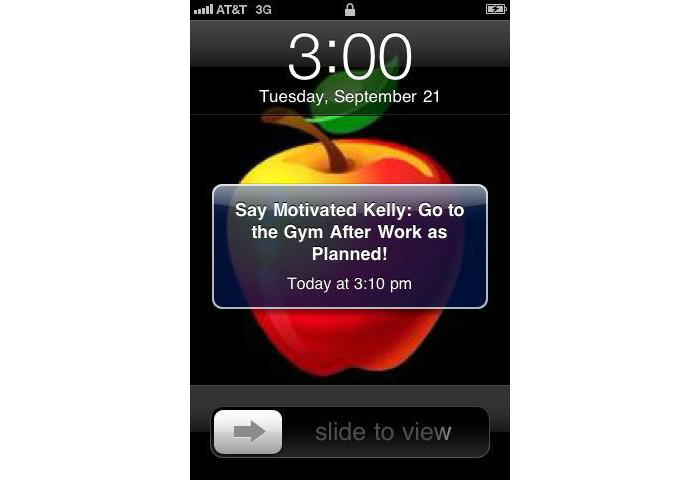
Tailored prompting to facilitate planned behavior.

The Health-E-Call application developed by the authors allowed participants to monitor up to 3 additional, weight-related behaviors (Figure 2) that were completely personalized. Typically, the behavioral targets for personalized self-monitoring were selected to overcome a barrier to weight loss (eg, preparing a healthy lunch before leaving home for work). Participants also received tailored prompts consisting of a brief message and an audible tone at the times that were most relevant to the targeted behavior (eg, shortly before leaving for work). Participants were able to create these optional behavioral targets, and determine the timing for prompts (Figure 3), with the help and approval of a study interventionist during the weekly weigh-ins described below.

**Figure 4:**
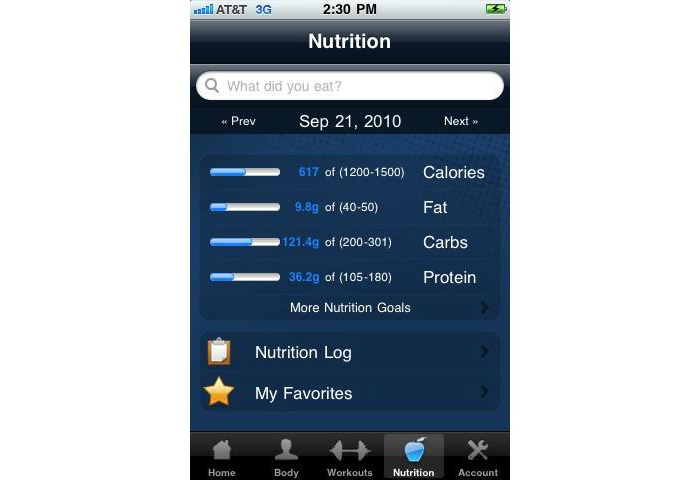
DailyBurn feedback on dietary intake.

A combination of automated and human feedback was provided to participants via their smartphones. DailyBurn provided automatic feedback on the number of calories consumed relative to the participants’ daily goal each time food intake was recorded (Figure 4). Weight was entered daily and the application provided graphed feedback of participants’ weights relative to their weight loss goals. DailyBurn provided a visual tally of the number of days that participants met their calorie and physical activity goals each week. Brief messages from a study interventionist were sent to participants’ smartphones 1-3 times per week by text messaging. This feedback was based on participants’ self-monitoring data, which was available to the study team in real-time due to the smartphones’ uninterrupted Internet connection. This feedback was primarily supportive and sometimes included tips for modifying eating and/or physical activity behaviors. While participants were able to send a text message in response to the feedback, they were not allowed to engage in a dialogue with the interventionist via text messaging.

The Health-E-Call application also provided access to 15 brief video lessons lasting approximately 5 minutes each, created by the researchers (see Multimedia Appendix 1 for an example). Each video was organized into one of the following topics: (1) Keeping Track, (2) In the Moment, (3) Planning Ahead, and (4) General Information. “Keeping Track” videos contained instructions on the use of DailyBurn and the investigator-developed self-monitoring tools. “In the Moment” videos provided skills training and behavioral recommendations for coping with immediate weight loss barriers (eg, eating in restaurants, coping with emotions, low motivation to be physically active). “Planning Ahead” videos included instructions for behavioral approaches that could facilitate healthy eating and physical activity habits in the future (eg, suggestions for grocery shopping). “General Information” videos provided education on topics such as “What is a calorie?” and “Adding Variety to Your Physical Activity Routine.”

The in-person treatment component began with an individual 60-minute session that was used to set goals for weight loss, caloric intake, fat intake, and time spent in structured physical activity. Participants were then trained in the use of the smartphone-based intervention system described above. For 12-weeks thereafter, participants attended weekly weigh-ins of 5-15 minutes with a study interventionist. These sessions were used to obtain an objective measure of body weight and address any challenges participants encountered while using the smartphone intervention system.

Paper lessons on behavioral weight loss topics (eg, choosing healthy foods, suggestions for physical activity, stimulus control, relapse prevention) were provided to participants during their weekly weigh-ins during the first 12 weeks of treatment, but not the second 12 weeks of treatment. Participants were encouraged to review these handouts on their own as the contents of the handouts were not reviewed during weigh-ins.

The contents of the smartphone-based videos and weight loss lessons were based on the approached used in the DPP and LookAHEAD [6,7]. Participants were encouraged to consume a low-calorie low-fat diet, engage in regular leisure time physical activity, and self-monitoring these behaviors as well as daily body weight. Participants set goals to lose at least 10% of their initial body weight during the first 12-week period, at a rate of approximately 0.5-1.0 kg (1-2 pounds) per week. They were given a calorie goal ranging from 1200 to 1800 kcal/day depending on their baseline weight, and were encouraged to consume no more than 30% of their diet in the form of fat. Participants were encouraged to gradually increase their time spent in moderate intensity physical activity to reach a goal of at least 200 minutes of moderate intensity physical activity weekly by the end of the 12-week program. Participants were encouraged to spread their weekly physical activity over at least 5 days, and to accrue their physical activity in bouts of at least 10 minutes. Brisk walking was recommended as the primary form of physical activity. While there was some overlap in the content of the paper and video lessons, the paper lessons tended to include more general information and education (eg, healthy vs unhealthy sources of dietary fat, the benefits of self-monitoring) while the video lessons provided more specific and targeted suggestions (eg, how to identify and remove high-fat items in cupboards, and how to record composite foods in the smartphone diary).

### Measures

Weight was measured in kilograms using a digital scale at baseline and every week of the 12-24 week program. Height was measured in centimeters at baseline using a stadiometer. Apprehension at using technology was measured at baseline using the 9-item Technology Anxiety scale (scores ranged from 7-63; higher values represent greater anxiety, [35]). Adherence to the self-monitoring protocol was recorded by the DailyBurn application. Participants were considered adherent on days they recorded their weight and had either 3 or more meals or food equaling 50% or more of their caloric goal for the day. Participants were considered non-adherent on days these criteria were not met (a similar approach has been used previously, [33]). In addition to these measures of adherence, DailyBurn recorded the average number of days per week physical activity was reported, and the average number of weekly physical activity minutes. The Health-E-Call application recorded the number of logins, videos viewed, and the use frequency of the personalized behavorial monitoring feature. The number of interventionist feedback messages provided to participants was recorded and a weekly average was calculated. Use of supplementary paper lessons on weight loss was not assessed. At the end of each 12-week treatment period, participants answered 2 questions on a 7-point likert scale to indicate their overall satisfaction with the weight loss program (very dissatisfied to very satisfied) and whether they would recommend the program to their friends, family, or coworkers (definitely would not recommend to definitely would recommend). Higher scores represented higher satisfaction and a greater likelihood of recommendation.

### Statistical Analysis

PASW Statistics 19 was used for all analyses. Descriptive statistics were generated for baseline demographic characteristics and outcome variables (weight loss, adherence to the self-monitoring protocol, physical activity reporting, use of the personalized behavioral monitoring feature, treatment satisfaction, and treatment session attendance) including means and SE for continuous variables and counts with percentages for categorical variables. Primary endpoints for the analysis of weight loss, were at the end of the 12- and 24-week weight loss phase. Correlations were used to test for associations between adherence and weight loss.

## Results

The 20 participants’ baseline characteristics are reported in Tables 1 and 2. On average, the participants were obese at baseline with a mean BMI of 36.3 kg/m^2^ (SE 1.2 kg/m^2^). Most participants (16/20) were provided with an iPhone 3GS for use in the study, but 2 participants acquired their own device during the trial and chose to use it instead for the remainder of their participation. All participants completed the initial 12-week program. Fifteen participants chose to continue treatment for an additional 12 weeks and all of these individuals completed the extended treatment. Of the 5 participants who chose not to continue, 3 agreed to be assessed at 24 weeks (2 reached their weight loss goal and reported feeling that further treatment was not necessary; 1 reported no desire to making further changes to her eating and activity habits), and 2 declined to be assessed at 24 weeks (1 participant was diagnosed with a serious medical condition unrelated to body weight at 12 weeks and was unable to continue treatment; 1 reported no desire to making further changes to her eating and activity habits).

Weight loss and body weight at baseline, 12 weeks, and 24 weeks are reported in Table 2. At 12 weeks, 85% (17/20) of participants lost at least 5% of their initial body weight and 40% (8/20) lost at least 10% of their initial body weight. At 24 weeks, 100% (15/15) of the participants who completed an additional 12 weeks of treatment lost at least 5% of their initial body weight and 87% (13/15) lost at least 10% of their initial body weight. Among the total sample, with 12-week values carried forward for participants who were not assessed at 24 weeks, the proportion of participants who reached the 5% and 10% weight loss milestones at 24 weeks was 90% (18/20) and 70%. (14/20).

The average Technology Anxiety Scale score at baseline was 20.3 (SE 2.6), with a range of 9-46 (min/max was 9/63). Baseline Technology Anxiety Scale scores were not associated with weight loss at 12 weeks (*r*=.102, *P*=.67) or 24 weeks (*r*=-.305, *P*=.19).

Adherence to the treatment protocol was measured by attendance at treatment sessions, number of days adherent to self-monitoring (ie, recording daily body weight and at least 3 meals or food intake per day equivalent to 50% or more of the daily calorie goal), and viewing of video lessons. Participants attended 91.7% (SE 2.2%) of treatment sessions during the first 12 weeks and 88.9 (SE 3.3%) of sessions during the second 12 weeks (including only those who received a second 12 weeks of treatment). On average, participants were adherent to the self-monitoring protocol on 90.8 (SE 3.3%) of days during the initial 12-week treatment period. Adherence during the second 12-week period was 84.9 (SE 4.0%, including only the 15 participants in the extended treatment program). Adherence to the self-monitoring protocol was correlated with weight loss (% of initial body weight) at 12 weeks (*r*=.47, *P*=.04), but not 24 weeks (*r*=.42, *P*=.124). The non-significant results at 24 weeks might be attributed to insufficient power due to the smaller sample size at 24 weeks (n=20 at 12 weeks vs n=15 at 24 weeks). On average, participants viewed 8.3 (SE 5.2) videos during the initial 12-week treatment period, and 3.1 (SE 2.1) videos during the second 12-week treatment period. The number of video lessons viewed was not associated with weight loss (*P*’s>.50).

Other factors related to engagement with the smartphone intervention and performance of weight loss behaviors included the reporting of physical activity minutes via DailyBurn, logins to the Health-E-Call application, and the use of the personalized goal-setting feature. Participants reported engaging in physical activity on 2.6 (SE 0.1) days per week for an average of 125.1 (SE 10.8) minutes per week during the first 12 weeks, and 2.9 (SE 0.2) days per week for an average of 140.7 (SE 12.3) minutes per week during the second 12 weeks. Participants accessed the Health-E-Call application on 3.4 (SE 0.2) days per week during the first 12 weeks and 3.1 (SE 0.2) days per week during the second 12 weeks. During the first 12 weeks, 6/20 (30%) participants used the personalized behavioral monitoring feature while 7/20 (47%) participants used the feature during the second 12 weeks. Of the 15 participants who completed the 24-week program, 3 (20%) used the behavioral monitoring feature (1 during weeks 1-12 only, 1 during weeks 13-24 only, and 1 during both 12-week periods). Participants who used this tool during the first 12 weeks used it for 1-4 weeks (mean 2.5, SE 0.5). During the second 12 weeks, the range was 1-6 weeks of use (mean 2.9, SE 0.7).

Participants rated their overall satisfaction with the program, and the likelihood that they would recommend the program to others, on a scale of 1 to 7. At 12 weeks, all participants but one (who rated satisfaction at 6) gave the maximum rating for satisfaction, and all participants gave the maximum rating for the likelihood that they would recommend the program to others. Of the 15 participants who completed the extended program, all participants endorsed the maximum rating for satisfaction and the likelihood that they would recommend the program to others.

**Table 1:** Participants’ characteristics (N=20).

Characteristic		n (%) or mean (SE)
**Gender, n (%)**		
	Women	19 (95)
Age in years, mean (SE), y		53.0 (1.9)
**Ethnicity, n (%)**		
	White (Non-Hispanic)	17 (85)
	African American	1 (5)
	American Indian	1 (5)
	Other	1 (5)
**Marital status, n (%)**		
	Single	2 (10)
	Married	11 (55)
	Separated/Divorced	7 (35)
**Education, n (%)**		
	High school or less	3 (15)
	Some college	5 (25)
	College or University Degree	7 (35)
	Graduate degree	5 (25)
Technology anxiety, mean (SE)		20.3 (2.6)

**Table 2:** Changes in participants’ weight at 24 weeks.

		Baseline mean (SE)	12 weeks mean (SE)	24 weeks mean (SE)
**Body weight (kg)**				
	24-week program completers (n=15)	97.4 (3.6)	88.5 (3.1)	84.9 (3.2)
	24-week assessment completers (n=18)	97.6 (3.5)	88.6 (3.0)	85.7 (3.2)
	Total sample (n=20)	95.8 (3.4)	87.4 (2.8)	84.9 (3.0)^a^
**Weight loss (kg)**				
	24-week program completers (n=15)	-	8.9 (0.8)	12.5 (1.0)
	24-week assessment completers (n=18)	-	9.0 (0.8)	11.9 (0.9)
	Total sample (n=20)	-	8.4 (0.8)	10.9 (1.1)^a^
**Weight loss (% of intial weight)**				
	24-week program completers (n=15)	-	9.4 (0.6)	12.8 (0.8)
	24-week assessment completers (n=18)	-	9.1 (0.6)	12.2 (0.8)
	Total sample (n=20)	-	8.5 (0.7)	11.2 (1.0)^a^

^a^12-week values carried forward for the 2 participants without data at 24 weeks

## Discussion

### Findings and Conclusions

This study was one of the first to use sophisticated smartphone technology to enhance self-monitoring and delivery of empirically validated BWL content and interventionist feedback in a formal weight loss program. The achieved weight loss, adherence to the study protocol, and study retention were excellent and compare favorably to the outcomes observed in prior trials of BWL delivered in group and individual treatment sessions [5]. Retention was 100% (20/20) for the initial 12-week treatment program, and engagement with the smartphone-based resources was high. The average 12-week weight loss exceeded 8% (SE 0.7) of initial body weight. The weight loss results at 24 weeks were similarly favorable, with the average weight loss exceeding 13% (SE 0.8) of initial body weight for treatment completers, which is unusual for a BWL, especially one of such short duration [5]. The weight loss obtained in this trial were substantially larger than the loss of 3-5% of initial body obtained in text message-based interventions [22,23].

Self-monitoring has been highlighted to be the “cornerstone” of BWL [36]. In this pilot study, adherence to the self-monitoring protocol was approximately 91% (SE 3.3%) and 85% (SE 4.0%) at 12 and 24 weeks, respectively. This was substantially higher than rates seen in other trials of BWL using paper diaries (eg, 55%, [33]). This finding is particularly remarkable because, unlike the electronic diary used in this study, paper diaries are often completed retrospectively, which can inflate estimates of adherence and may negate much of the benefit of self-monitoring [37]. The high levels of adherence to self-monitoring in Health-E-Call likely contributed to favorable weight loss outcomes, as seen by the significant correlation with weight loss. The high rates of adherence in this study are attributable to several factors associated with the smartphone-based approach, such as ease of use, and the immediacy of feedback, which may have increased engagement. Participants were also aware that study staff could monitor their adherence to the self-monitoring protocol in real-time and prompted adherence when a lapse was noted. This extra accountability and support, which was not possible in traditional BWLs using paper diaries, also likely contributed to improved adherence.

The personalized behavioral monitoring feature was a novel and unique aspect of the smartphone-assisted intervention. Participants were encouraged to use this tool creatively and the outcome was highly idiosyncratic in both the behavioral targets and the strategy of use. Participants who used the tool commonly set one or more standing goals to facilitate the development of a new healthy habit (eg, going to the gym after work, refusing high calorie food routinely offered by a friend or family member, eating 5 servings of fruit and vegetables daily) over one or more weeks. Some participants kept the same goal(s) for multiple weeks while others changed goals routinely. Some also used the tool to send themselves encouraging and supportive messages at times of the day or week when they often experienced challenges to their healthy eating or physical activity behaviors (eg, “Remember that you are in control of your eating!”, or “If you’re feeling stressed, there are other ways to cope besides eating”). The personalized behavioral monitoring feature was also sometimes used to prompt a one-time behavior such as buying a piece of exercise equipment or asking for a family member’s support with the weight loss effort. Notably, 65% (13/20) of participants did not use this feature, primarily because they were able to reach their weight loss goals without it. Thus, future research should test the efficacy of personalized behavioral monitoring, and for whom and under what conditions it is most beneficial.

The positive outcomes of this pilot study may be due, in part, to the intensive nature of the intervention. The effect of the smartphone-based resources cannot be disentangled from the effects of other intervention components. The intention with this pilot study was to ensure that participants received sufficient contact with the research team to ensure they were able to follow the study protocol as intended. When an established treatment is translated to a new delivery modality, it may be desirable to make the transition in a series of steps that provide the opportunity to understand how best to make the transition, and reduce risk that efficacy will be substantially impaired due to unforeseen challenges with the new modality. This was felt to be particularly important in Health-E-Call, given that many participants had very little prior experience or comfort using smartphones or other forms of technology. In actuality, none of the participants required special coaching in the use of the smartphone apps beyond what was planned in the protocol, and weight loss was not associated with comfort using technology. Thus, the frequency of in-person contact should be reduced in future studies using this approach. While contact with a human interventionist may improve outcomes in electronically-delivered weight loss treatments, randomized clinical trials are needed to identify the optimal rate of contact that balances weight loss outcomes with interventionist time and cost.

### Limitations and Future Directions

The small, homogenous, sample was a limitation of this study, as was the lack of a control group or comparison condition. It is also important to acknowledge that providing a smartphone to 16/20 participants may have positively influenced retention and adherence to the self-monitoring protocol. Lastly, participants were not followed after 24 weeks of treatment, and the long-term effects of the treatment are unknown. Despite these limitations, this study was important because it was one of the first to test a sophisticated smartphone-based system for BWL delivery, with very favorable weight loss outcomes, and very high rates of compliance with the self-monitoring protocol. Future research should be conducted to test this novel treatment in a larger randomized controlled trial.

## Multimedia Appendix 1

Example intervention video available within the Health-E-Call smartphone application.

## Abbreviations

BMI: body mass index
BWL: behavioral weigh loss treatment
DPP: Diabetes Prevention Program
LookAHEAD: Action for Health in Diabetes
WCDRC: Weight Control and Diabetes Research Center
